# A simplified seasonal forecasting strategy, applied to wind and solar power in Europe

**DOI:** 10.1016/j.cliser.2022.100318

**Published:** 2022-08

**Authors:** Philip E. Bett, Hazel E. Thornton, Alberto Troccoli, Matteo De Felice, Emma Suckling, Laurent Dubus, Yves-Marie Saint-Drenan, David J. Brayshaw

**Affiliations:** a Met Office Hadley Centre, FitzRoy Road, Exeter EX1 3PB, UK; b School of Environmental Sciences, University of East Anglia, Norwich NR4 7TJ, UK; c World Energy & Meteorology Council, University of East Anglia, Norwich NR4 7TJ, UK; d ENEA, Bologna, Italy; e Department of Meteorology, University of Reading, Reading RG6 6BB, United Kingdom; f National Centre for Atmospheric Science, University of Reading, Reading, United Kingdom; g EDF R&D, OSIRIS Dept, 7 boulevard Gaspard Monge, 91120 PALAISEAU, France; h MINES ParisTech, PSL Research University, O.I.E. Centre Observation, Impacts, Energy, 06904 Sophia Antipolis, France

**Keywords:** Seasonal forecasting, Renewable energy, Wind, Solar, Climate services

## Abstract

We demonstrate levels of skill for forecasts of seasonal-mean wind speed and solar irradiance in Europe, using seasonal forecast systems available from the Copernicus Climate Change Service (C3S). While skill is patchy, there is potential for the development of climate services for the energy sector. Following previous studies, we show that, where there is skill, a simple linear regression-based method using the hindcast and forecast ensemble means provides a straightforward approach for producing calibrated probabilistic seasonal forecasts. This method extends naturally to using a larger-scale feature of the climate, such as the North Atlantic Oscillation, as the climate model predictor, and we show that this provides opportunities to improve the skill in some cases.

We further demonstrate that, on seasonal-average and regional (e.g. national) average scales, wind and solar power generation are highly correlated with single climate variables (wind speed and irradiance). The detailed non-linear transformations from meteorological quantities to energy quantities, which are essential for detailed simulation of power system operations, are usually not necessary when forecasting gross wind or solar generation potential at seasonal-mean regional-mean scales.

Together, our results demonstrate that where there is skill in seasonal forecasts of wind speed and irradiance, or a correlated larger-scale climate predictor, skilful forecasts of seasonal mean wind and solar power generation can be made based on the climate variable alone, without requiring complex transformations. This greatly simplifies the process of developing a useful seasonal climate service.

## Highlights

• The skill in seasonal forecasts of wind and irradiance in Europe is patchy.
• A linear regression method can produce calibrated probabilistic seasonal forecasts.
• Seasonal + regional average wind/solar power is highly-correlated with wind/irradiance.
• So, detailed transformations (power curves etc.) are not necessary at these scales.
• Together these results simplify the production of seasonal climate services.

## Graphical abstract

**Figure:**
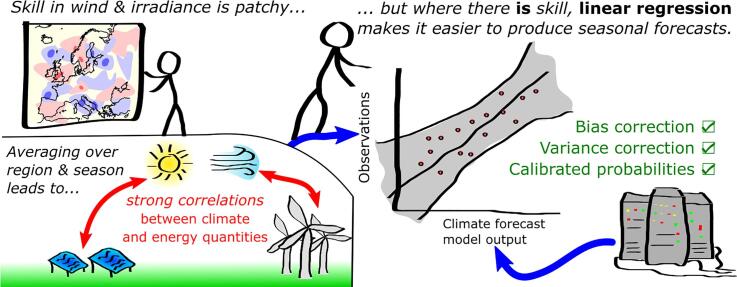

**Practical Implications**

There is an increasing demand for seasonal climate prediction services for the energy sector, in order to improve system resilience, energy security, financial planning or to reduce financial risks. Potential users include power plant managers and operators (e.g. wind/solar farms), distribution or transmission system operators, regulators, policy makers and financial traders.

Greater availability of seasonal forecast and hindcast data, through projects like the Copernicus Climate Change Service (C3S), is enabling many organisations – private companies, national meteorological services, energy companies – to start to develop seasonal climate services for their customers’ needs.

We show that the forecast skill for seasonal mean wind speed and solar irradiance in Europe, at 1-month lead times, is very patchy: although it is high enough to be useful in some cases, this is far from universal across all regions and seasons. Services should be developed for specific applications, for specific regions and seasons rather, than as a generic tool.

We demonstrate that a simple methodology, based on linear regression between a climate model predictor variable and the observed variable of interest to the users, can greatly simplify the production of a calibrated probabilistic seasonal forecast.

We also show that, for seasonal and regional averages (e.g. over a European country), wind and solar photovoltaic (PV) power generation are both very highly correlated with the average wind speed and solar irradiance, respectively. Given the level of uncertainty in seasonal forecasts, and the modest levels of forecast skill, this limits the benefit from complex transformations performed at high temporal/spatial resolution, such as wind turbine power curves, scaling to turbine hub height, or including the temperature-dependence of solar PV power.

This methodology requires the availability of multi-decadal time series of the quantity of interest, for example wind power, to calibrate the forecasts. Suitable data will often not be available directly, requiring an additional calibration step to relate recent electricity production to observed climate variability over a longer period. In any case, close collaboration with the prospective user will ensure the seasonal forecast service is targeted at the most relevant quantity. A service might also be further improved through direct engagement with climate forecast data providers, to utilise the latest models, data sets and research into how to optimise the use of the seasonal forecasts.

## 1 Introduction

Seasonal climate prediction, in which statistics of the weather over a period of several months, are forecast with a lead time of several weeks, has long been an area of interest to the energy sector (e.g. Weiss, 1982, Troccoli et al., 2008, Troccoli, 2010, Doblas-Reyes et al., 2013). Recent improvements in the levels of skill in seasonal forecast systems, particularly at mid-latitudes (e.g.Scaife et al., 2014, Smith et al., 2016), have meant that seasonal forecasting climate services are now starting to be developed in earnest (e.g. Palin et al., 2016, Viel et al., 2016, Prudhomme et al., 2017, Clark et al., 2017, Buontempo, 2018, Thornton et al., 2019). At the same time, the introduction of increasingly important levels of weather-dependent renewable electricity generation means that demand for skillful and reliable seasonal forecasting services, tailored to the requirements of users in the energy sector, is only likely to increase in the coming years.

The energy sector is itself very diverse, particularly when considering the different arrangements across European countries: owners and operators of electricity generation facilities, operators of the transmission or distribution networks, energy traders, system regulators and policy makers all have different needs and aims in terms of climate services. Indeed, such organisations often employ specialist meteorologists: they help to translate the weather and climate conditions in the forecasts, and their uncertainties, into the energy-related quantities required by their colleagues for decision-making. They therefore act as internal weather and climate service providers.

Increasing amounts of observational and forecast data are now being made more easily available to users, through initiatives such as the European Commission’s *Copernicus Climate Change Service* (C3S), in partnership with national meteorological services and other organisations across Europe. For example, the *European Climatic Energy Mixes* (ECEM) proof-of-concept service, a C3S Sectoral Information System, developed new observation-based data sets that are relevant for studying the impacts of climate variability on the European energy sector. It has also examined the skill of seasonal forecasts provided through the C3S Climate Data Store (Troccoli et al., 2018). However, a gap remains between developers of these kinds of data sets, and the needs of users within energy sector organisations. It is this gap that we target in this paper, by demonstrating how seasonal climate forecast data, made available through programmes like C3S, could be used to provide useful information for the energy sector.

A typical approach for producing a seasonal forecast for the energy sector (or other sectors) would start with the forecast ensemble of the meteorological variable, or variables, of interest. By analogy with the needs of short-term (“weather”) forecasts, these might be obtained at very high temporal resolution, to allow for a precise, non-linear transformation into the energy metric required. In addition to requiring bias and perhaps variance correction, the forecast ensemble is likely to require calibration to ensure that it produces reliable probabilistic forecasts, in the statistical sense: that is, whenever forecasts of particular conditions are made with a given probability, they should then occur with that frequency. This combination, of detailed non-linear transformations of high-frequency, possibly multi-variate data, all requiring probabilistic calibration, can make development of a seasonal climate service highly challenging; even if only from a data volume and computational perspective. In practice, this means that many in the energy sector base their assessments of future conditions on historical climatological data, rather than forecasts. Even when seasonal climate forecasts are used, it tends to be qualitatively rather than quantitatively.

In this paper, we use data produced in the ECEM project to demonstrate how seasonal forecasts for the European wind and solar energy sectors, particularly seasonal-mean, regional-mean forecasts of meteorological or energy quantities at 1-month lead times, can be produced in a much more straightforward way, without compromising the need to provide probabilistic information. Note that our focus is on the methodology, rather than the specific results in any individual case.

In Section 2, we describe the seasonal hindcast and observation-based data sets we use to assess the forecast systems. We then consider the skill of these systems in forecasting seasonal mean wind speed and irradiance in Section 3, and demonstrate a simple approach for producing calibrated probabilistic forecasts. Section 4 describes how we might translate the skill found in forecasting climate variables into skillful forecasts of potential wind power and solar power generation. We discuss the benefits of more detailed co-design of forecasting services in Section 5. Finally, we summarise our conclusions in Section 6.

## 2 Data sets

To assess the performance of different seasonal forecast systems, we use their hindcast data sets, obtained from the C3S Climate Data Store. We compare the hindcasts against observation-based data sets, including those produced through the ECEM project. We describe these in the following subsections.

### 2.1 Seasonal hindcasts

Three hindcast data sets were obtained from ECMWF during the pre-operational phase of the C3S Climate Data Store (Raoult et al., 2017)^2^ in late 2017, from three different production centres: ECMWF (Molteni et al., 2011), Météo-France (Météo-France, 2015) and the Met Office (MacLachlan et al., 2015, Williams et al., 2015). Table 1 describes some key details of these three forecast systems, relevant for the present study. The forecast systems differ not only in the formulation of their underlying climate models, but also in the way the forecasts are initialised, and in how the forecast and hindcast data sets are compiled from those initialised runs. We refer the reader to the references above for more comprehensive descriptions of each particular system.

**Table 1:** Summary details for seasonal prediction systems used here. The years in the hindcast period column refer to those of the initialisation dates (May and November). The Forecast System column refers to the version numbers assigned by the C3S Climate Data Store. All data is regridded to a 1° grid before use.

**Production centre**	**Forecast** System	**Model**	**Spatial** resolution	**Hindcast** period	**Hindcast** ensemble
ECMWF	System 4	IFS Cyc36r4	T255 L91 (∼ 80 km)	1981–2010 (30 years)	51 members
Météo-France	System 5	Arpege-IFS Cyc37	T255 L91 (∼ 80 km)	1993–2014 (22 years)	15 members
Met Office	System 12 (GloSea5)	HadGEM3-GC2	N216 L85 (∼ 60 km)	1993–2015 (23 years)	28 members

Each hindcast comprises an ensemble of climate model simulations that are run forward for several months after initialisation. A new, independently initialised set of runs is available for every month of each 20–30 year data set. This allows the behaviour of each forecast system to be examined by providing a series of retrospective climate predictions. Although these large data sets provide the freedom to examine forecasts of many different periods over a range of different lead times, seasonal forecasts typically focus on forecasting for 3-month seasons, with a lead time of about one month. Here, we consider forecasts of the average conditions in winter (December–January–February, DJF) and summer (June–July–August, JJA), initialised in early November and May respectively.

### 2.2 Observation-based data

We use two reanalysis data sets as proxies for observations of climate data. Primarily, we use ERA-Interim reanalysis data (Dee et al., 2011), covering the period 1979–2016 at 0.75° grid resolution. We also use the climate data set that was developed as part of the ECEM project (Jones et al., 2017), which is based on ERA-Interim, but bias-adjusted using various station-based and satellite-based observational data sets. Both data sets were regridded to a 1° grid before use, for comparison with the hindcasts. The ECEM climate data is also available as national averages, making it easier to compare with the energy data.

As a proxy for the observed levels of wind and solar PV electricity generation, we use the national-scale energy data sets developed in ECEM (Dubus et al., 2017a, Dubus et al., 2017b, Saint-Drenan et al., 2018; see also Troccoli et al., 2018). While this is based on actual, observed generation data from across Europe, it is in fact modelled. The capacity factor (the amount of power generation at a given moment as a fraction of the installed generation capacity) for a given generation source, such as wind, is modelled and calibrated against measured data over a recent period with a known installed capacity. This model is then applied back over the historical period, driven by the ECEM climate data, while imagining the same installed capacity as in the present. This allows the production of long time series that accurately describe the meteorological dependence of electricity generation in different regions. Without this, the data would be dominated by the varying technological, economic, political or social factors that strongly affect the actual levels of installed capacity, which vary markedly over time. Although the data is also provided in terms of total generation (i.e. energy) and mean generation rate (i.e. power) as well as capacity factors, we simply use the capacity factor data here as it is not necessary to convert further for our analysis.

The national-scale ECEM climate and energy data sets cover 33 European countries (23 for wind power). An important restriction is that offshore areas belonging to countries are excluded, as much of the underpinning ECEM climate data was bias adjusted using measurements from land stations.^3^ Offshore wind power generation has much higher capacity factors than onshore, and some countries have significant amounts of offshore wind power installed. The energy results therefore shouldn’t be seen as reflecting the true “national” capacity, but the land-based capacity. However, this does not affect our main points regarding methodology.

We use the ECEM wind power data that is based on a statistical model using a support vector regression technique. A lack of adequate training data in some cases means that it only covers 23 countries, although it tends to perform slightly better than the ECEM data produced using a physically-based wind turbine model (see Dubus et al., 2017b for details). In practice, they are both well correlated and the choice does not affect our results (Bett et al., 2018a).

The solar photovoltaic (PV) generation data from ECEM is based on the mixed physical and statistical method of Saint-Drenan et al. (2017). It takes into account the tilt and orientation of the solar panels, and includes a dependence on air temperature as well as irradiance to estimate power output for a reference PV system (solar PV panels operate more efficiently at lower temperatures).

The detailed formulation of the models for wind and solar power is not the focus of this study, and indeed many model variations were tested as part of the ECEM project. The strength of the resulting data sets lies in them covering the same multi-decadal period, having been calibrated against a comprehensive set of national electricity production data gathered from a range of sources. We shall be treating them as the observational “truth” for the purposes of this study.

## 3 Calibrated probabilistic forecasts of climate variables

In this section we describe the skill of the three forecast systems in predicting mean 10 m wind speed and irradiance, and demonstrate how the ensemble means can be used to provide calibrated probabilistic forecasts of these quantities.

### 3.1 Skill of direct forecasting of climate variables

**Fig. 1:**
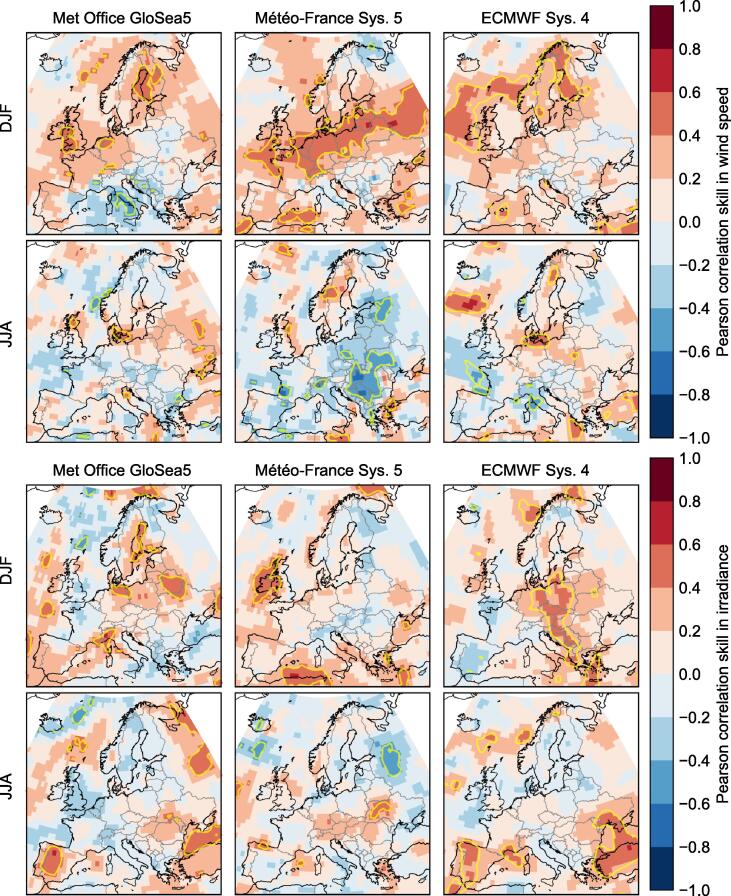
Skill, as measured by the correlation coefficient, of seasonal forecasts of 10 m wind speed (upper rows) and irradiance (lower rows), from the three hindcasts we use here (columns, as labelled), against ERA-Interim data. Forecasts are for the 3-month averages of winter (DJF) and summer (JJA) as labelled, at a lead time of one month (i.e. November and May initialisation respectively). The yellow contour marks a notional threshold for significance, using the Fisher z-test at the 5% level.

One of the simplest ways of measuring the forecast skill of a given variable is through the interannual Pearson correlation between the observed values and the hindcast ensemble-mean values. The correlation skill for wind speeds and irradiance, for the three forecast systems in both summer and winter, is mapped in Fig. 1. (There is negligible difference if using the ECEM climate data instead of ERA-Interim.).

The skill is clearly patchy, and varies between the different models, seasons and variables: one cannot make broad statements like “model X has skill in forecasting variable Y”. This is typical of seasonal forecasting in mid-latitude regions, and is important for informing expectations about seasonal forecasts, such as when communicating with potential users: seasonal forecasts perform at a very different level of predictability than traditional weather forecasts, or even medium-range subseasonal ensemble forecasts. They must be used selectively, choosing only the cases (regions, seasons, models, variables) where we can be confident that there is skill.

Furthermore, since the correlation is based on the very limited number of years in the hindcast data sets, it is itself rather uncertain. A confidence interval on the correlations can be calculated using a Fisher z-transformation. This is a simple analytic estimate, which assumes that the hindcast and observational data follow a bivariate normal distribution. While this is clearly not true for wind speed and irradiance in general (e.g. winds are often considered to follow a Weibull distribution: Hennessey, 1977, Carta et al., 2009, Harris and Cook, 2014), it is a reasonable assumption in this case because of the Central Limit Theorem: after averaging to get seasonal means, country means and ensemble means, the remaining 20–30 pairs of data points are usually indistinguishable from being normally distributed. The correlation values for the confidence interval are given by

(1) $$r_{\mathrm{CI}\pm }=\tanh\left(\mathrm{artanh}(r)\pm \frac{z_{2.5}}{\sqrt{N-3}}\right),$$

where *r* is the correlation whose confidence intervals we are estimating, and $z_{2.5}$ is the value at the 2.5th percentile of a standardised normal distribution, such that the confidence interval on the correlation is at the 95% level. Note that this confidence interval depends only on the number of years *N* in the data sets, and the value of the correlation itself. This means that we can write down the critical correlation thresholds for significance by this measure, $r_{\mathrm{crit}}$ (the smallest correlation *r* such that $|r_{\mathrm{CI}\pm }|>0$), which for the hindcasts we use here are:

• $r_{\mathrm{crit}}(N=30)=\pm 0.360$ (ECMWF)
• $r_{\mathrm{crit}}(N=23)=\pm 0.412$ (Met Office)
• $r_{\mathrm{crit}}(N=22)=\pm 0.422$ (Météo-France)

Contours marking the notional 5% significance thresholds on the correlations according to this test are marked on the skill maps in Fig. 1.

There is also uncertainty due to the finite ensemble size. However, due in part to the signal-to-noise problem (discussed in the next subsection), the skill increases systematically with ensemble size (e.g. Dunstone et al., 2016), following a clear theoretical relationship (Murphy, 1990). Furthermore, since the forecast ensembles are the same size or larger than the hindcast ensembles, it is safe to treat the skill we find here as a lower limit on the actual forecast skill, and we do not consider the impact of ensemble size further.

**Fig. 2:**
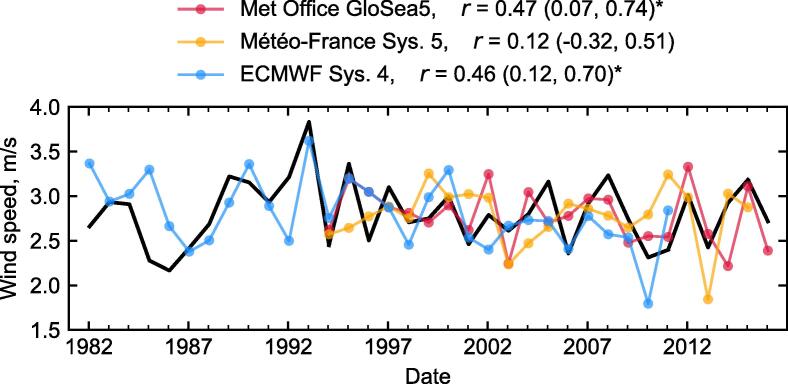
Time series of winter 10 m wind speed in Finland, showing observations (black) and hindcast ensemble mean data (colours, as labelled), after bias and variance correcting (see text). Points are plotted at the January of the DJF period they cover. The correlations *r* between observations and hindcast are shown in the legend, including their 95% confidence intervals in brackets. They are marked with a * where the correlation is significantly different to zero.

Area-weighted averaging over relatively large regions can enhance the forecast skill by reducing the gridpoint-scale noise. In Europe, individual countries can represent sufficiently large areas to achieve this, and often represent relevant administrative boundaries for users, making it a convenient choice for aggregating the forecasts. Time series of observations and hindcasts for each country are available on the ECEM Demonstrator.^4^ As this study focuses on methodology, we give an illustrative example in Fig. 2, showing hindcasts of winter wind speed in Finland from the three systems, together with observations. Because it is likely that some degree of bias and/or variance correction will always be necessary when working with climate model output, we show the hindcast ensemble means in Fig. 2 after applying a simple linear correction, which leaves the correlation skill unchanged:

(2) $$U_{\mathrm{hc}}^{☆}(t)=\overset{‾}{U_{\mathrm{ob}}}+\left(U_{\mathrm{hc}}(t)-\overset{‾}{U_{\mathrm{hc}}}\right)\frac{\sigma (U_{\mathrm{ob}})}{\sigma (U_{\mathrm{hc}})},$$

for seasonal mean wind speed data *U*, where the ☆ indicates the corrected data, the overbar indicates the long-term mean, σ is the interannual standard deviation, ‘hc’ indicates the hindcast ensemble means and ‘ob’ indicates the observation-based data. While it is important to understand any biases in the mean state or variability of the climate model, in order to improve the model and its forecasts, that is not our goal here: the important quantity in this case, in terms of skill, is the standardised co-variability of the initialised model with respect to the observations, i.e. the correlation.

### 3.2 Calibrated probabilistic forecasts based on ensemble means

The uncertainty of seasonal forecasts means that, in order to provide useful and robust information for decision-making, they should be used probabilistically. The simplest approach is to use the distribution of ensemble members directly as a description of the forecast probability distribution. However, there are many other methods of deriving probabilistic forecasts from the forecast ensemble, known in general as Ensemble Model Output Statistics (EMOS, e.g. Wilks, 2020). These might be preferable to avoid sampling error due to the finite (and historically small) ensemble size. A simple approach would be to appeal to the Central Limit Theorem again, and assume that the “true” forecast probability distribution is just a normal distribution with the mean and variance well estimated by those of the ensemble members. Other more precise techniques include forms of kernel dressing (e.g. Bröcker and Smith, 2008, Suckling and Smith, 2013, Smith et al., 2015).

A key requirement is that the probabilities generated by the forecast system are *reliable*, in the formal statistical sense: of the times when an event is forecast with a given probability (say, 70%), we should observe it to occur with the same frequency as that probability. If, when forecast, the events are observed to occur more frequently than that forecast probability, e.g. 90% of the time, then the forecasts are underconfident. Similarly, if the event occurs less often (e.g. 50% of the time), then the forecasts are overconfident. Just as forecasts will, in general, need some form of bias and variance correction, they will also need some degree of calibration to ensure they produce reliable probabilities.

Although climate predictions have often been found to be overconfident (i.e. ensemble members agree with each other better than they agree with the observations), it has recently been discovered (Eade et al., 2014, Scaife and Smith, 2018) that many climate models also produce *underconfident* forecasts in some cases. This particularly affects the North Atlantic sector, including dynamical features such as the North Atlantic Oscillation (NAO) and Arctic Oscillation (AO), which have a direct influence on features of the European winter climate such as wind speed (Clark et al., 2017). As discussed in the recent reviews of Scaife and Smith, 2018, Merryfield et al., 2020 and Meehl et al. (2021), and references therein, this underconfidence stems from the ensemble members exhibiting less predictable variability than the observed world. This means that we should be cautious of using the ensemble members to estimate forecast probabilities. Probabilistic methods can instead be developed based on the ensemble mean, as a quantity that maximises the skillful signals available from the climate model by reducing the noise from the individual members. The underconfidence implies that the ensemble mean anomalies will be too small, emphasizing the need for bias and variance correction. Having a large forecast ensemble will also improve the skill of the ensemble mean, as it will allow greater reduction of noise from the ensemble members.

All the approaches described above for producing probability distributions from the forecast ensemble would need probabilistic calibration, in addition to bias and variance correction of the mean. Various techniques have been devised to achieve this (e.g. Gneiting et al., 2005, Sansom et al., 2016, Torralba et al., 2017, and references therein). We will describe here a simple method of producing calibrated probabilistic forecasts, without using the ensemble member distribution at all, based on the traditional Model Output Statistics approach (MOS, Glahn and Lowry, 1972).

Rather than considering the observations and hindcast ensemble means as time series, we can instead examine their joint distribution. This can be shown as a scatter plot, which also directly illustrates their correlation. We can describe the linear relationship between the two data sets, as well its uncertainty, through a simple linear regression. If we then have a forecast of the predictor variable from the climate model, we can use the linear regression to transform it into a forecast of a future observation. The probabilities of any given value being observed subsequently are provided by the prediction interval on the regression.

**Fig. 3:**
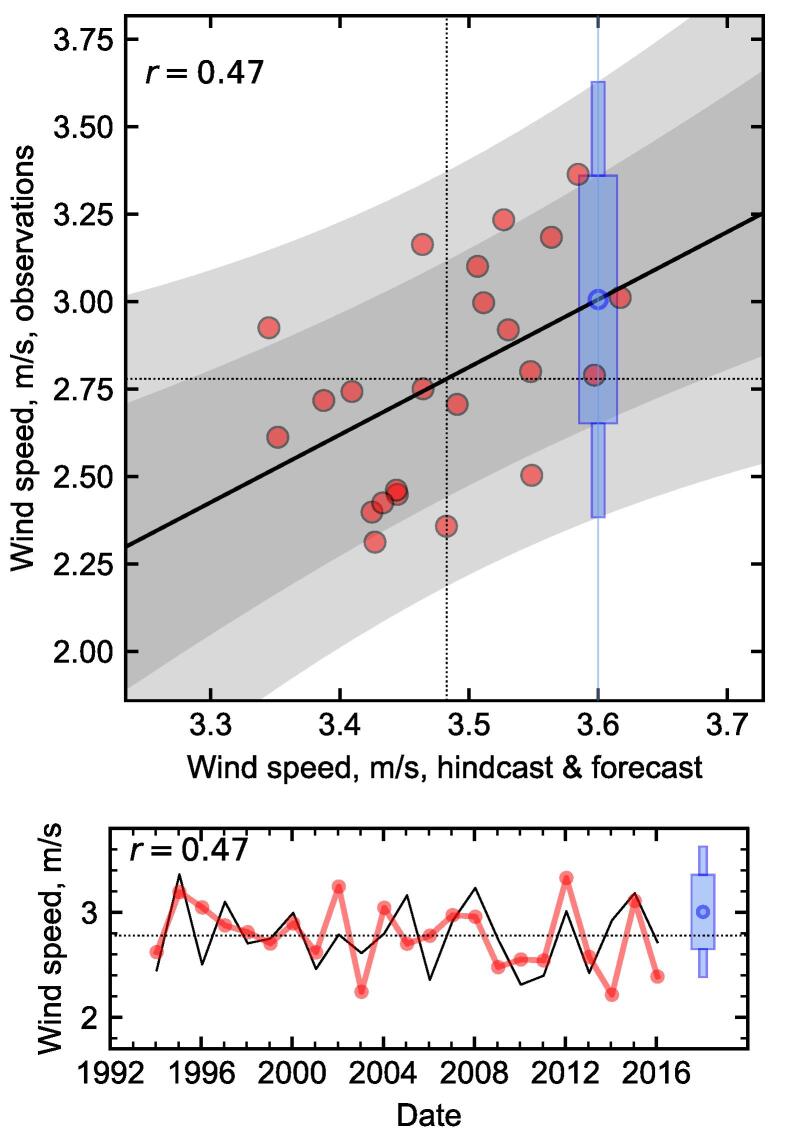
Winter mean 10 m wind speed in Finland, showing hindcast data from the Met Office system, and observations from the ECEM climate data. Top panel: Scatter plot showing the relationship between hindcast ensemble means and observations (red dots, one per year, shown without bias or variance correction). Their means are shown as horizontal and vertical dotted lines. The linear regression is shown as a black line, with the inner 75% and 95% of the prediction interval in grey shading. A hypothetical forecast is shown in blue at $3.6\;m\;s^{-1}$, with boxes highlighting the prediction interval at that point. Bottom panel: Time series display of the same data. The observations are in black, and the hindcast points (red) are plotted after bias and variance correction. The hypothetical forecast is shown again in blue.

We illustrate this procedure in Fig. 3, for the Met Office hindcasts of winter mean wind speed in Finland (data already shown in Fig. 2). In the scatter plot, the hindcast data are shown without bias and variance correction, to illustrate that this is taken care of by the linear regression. An imagined forecast is included, shown in blue, in which the climate model produced an ensemble mean forecast of $3.6\;m\;s^{-1}$. The plot then shows the central estimate of the predicted future observation, at approximately $3.0\;m\;s^{-1}$. The probability of the new observation being above average can also be seen: it is the fraction of the prediction interval that is above the dotted horizontal mean line. Because linear regressions are monotonic, this is the *only* point along the horizontal axis where the wind speeds are forecast to be above average with this probability; and the probability is given by the prediction interval, which *is* the conditional distribution of the observations given a forecast with that probability, taking the unavoidable sampling uncertainty into account. Therefore, as long as it is reasonable to describe the relationship between forecast and observations with a linear regression, then the resulting forecast probabilities are well-calibrated by construction, given the limitations of the data available. We give a more mathematical description of this point in Appendix A, with examples of reliability diagrams that explicitly demonstrate the calibration of hypothetical underconfident and overconfident forecasts. So, just as the linear regression bias-corrects and variance-corrects the hindcast data to match the observations, it also calibrates the probability distributions, such that they match the observed frequencies.

It is important to emphasise that this only applies because the system can reasonably be described by a linear model: the Central Limit Theorem, due to averaging over a season, region and the ensemble, pushes the two data sets towards being normally distributed, so that where there is good correlation skill then there will be a reasonably linear relationship. Where there isn’t a good correlation, then a linear model would have a null gradient, and the probability of any forecast will just be the frequency distribution of the observations: i.e. the climate model no longer contributes, and the forecast is given by the observed climatology. On the other hand, if we were not aiming to forecast an average quantity, for example if we are counting the occurrence of some event per season, then the Central Limit Theorem might not apply, and the data might not follow a linear relationship. In these cases, a different approach might be necessary, and this will be discussed in the next subsection.

It is also expected that, if orders of magnitude more data were available, such as centuries of points instead of decades, and if the skill was significantly higher, then there might be justification for using much more precise techniques to refine the probabilistic distribution (e.g. more detailed EMOS techniques, machine learning, etc.). However, as we have seen, seasonal forecast skill for wind and irradiance in Europe tends to be not much above the threshold for statistical significance at best, and there can only be limited benefit in more detailed statistical techniques – making precise fits to noise is unhelpful.

Fig. 3 shows the result of adding a new forecast point after the existing 23-year hindcast period. This reflects the procedure that would be used in a real-time forecast. However, it can also be helpful to understand the behaviour when using the same method to “forecast” any of those 23 historical years, in each case calculated with reference to the remaining 22 years only. This leave-one-out cross-validation procedure allows us to estimate the skill in forecasting years like those observed, and to understand the sensitivity of our method to outlier years. However, it is likely to yield lower values of skill, as each forecast is based on less data.

**Fig. 4:**
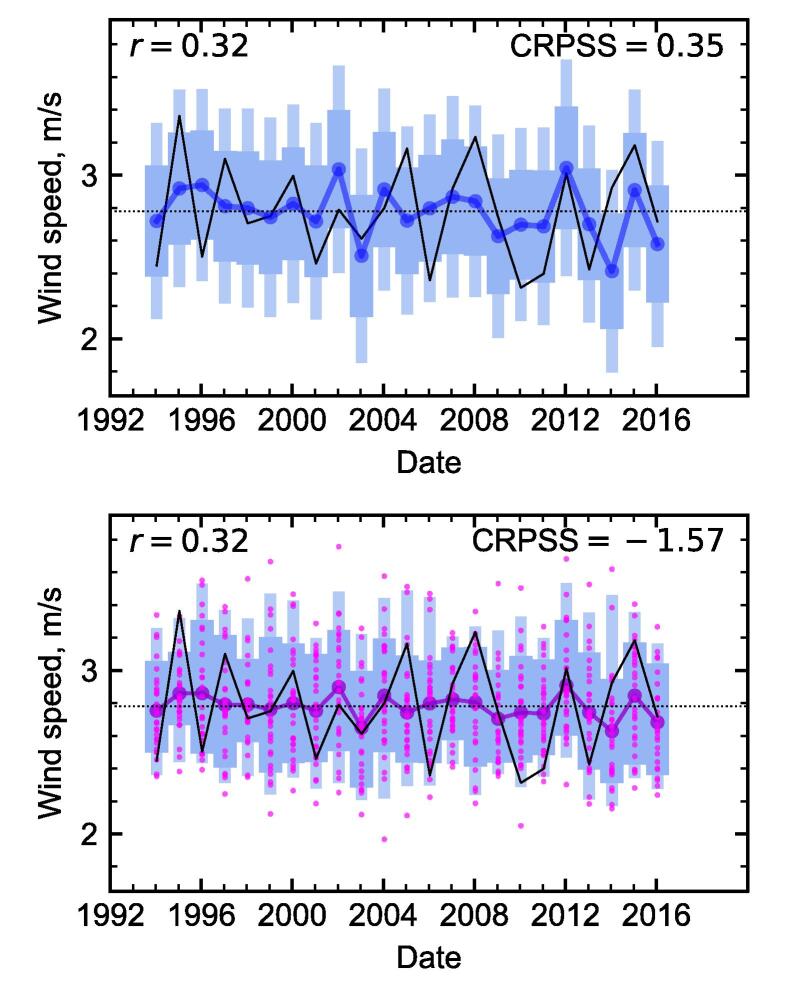
Leave-one-out cross-validated forecasts for the same data as Fig. 3, winter mean 10 m wind speed in Finland. Both panels use the same axis limits, and are and are labelled with the correlations *r* of the observations with the forecast central estimates, and the CRP skill scores from the forecast distributions. The time series of observations is shown in black, and the blue boxes show the inner 75% and 95% of the forecast probability distributions. Top panel: Forecasts based on linear regression. The boxes show the prediction intervals for each forecast, and the blue connected dots give the forecast mean. Bottom panel: The forecast ensemble members are shown by pink points, with the forecast ensemble means shown with larger purple connected dots. The blue boxes show percentiles of the ensemble distribution. Each ensemble mean was leave-one-out bias corrected, and the same correction applied to the ensemble member.s for each year.

Fig. 4 shows the results of leave-one-out cross-validation, and compares our linear regression approach with simply using the forecast ensemble. The correlation skill in both cases is the same by construction, as the linear regression is based on the hindcast ensemble means. The value of 0.32 appears lower than the 0.47 seen in Fig. 3, as expected given the reduced sample size. However, it is important to take the uncertainty into account when interpreting these values: the 95% confidence interval on the correlation of 0.47 is 0.07–0.74.

We have so far focused on the correlation skill of the ensemble mean, as this is directly related to our linear regression method. However, we can also assess the performance of the forecast probability distribution each year, for example using the continuous ranked probability score (CRPS, e.g. Hersbach, 2000, Wilks, 2020). This compares the forecast probability distribution with the observation each year, awarding higher skill (lower CRPS) when there is more probability closer to the observed value. We compare the mean score over all forecasts with the mean score from using the observed climatological mean as a (deterministic) forecast, to calculate a skill score (CRPSS). Positive values of CRPSS represent an improvement on climatology, with 1 representing a perfect forecast. In the limit of deterministic forecasts, the mean CRPS reduces to the mean absolute error. Fig. 4 shows the probability distributions from the linear regression have positive skill, with a CRPSS of 0.35. In contrast, the probabilistic skill is negative (worse than climatology) if the ensemble members are used alone.

The CRPSS is one of a wide range of probabilistic skill scores, and assesses the whole forecast distribution. Some users will be able to identify particular thresholds for dichotomous decision-making (e.g. if the wind does or does not exceed the observed upper tercile), and many probabilistic scores assess the likelihood of exceedance of these thresholds. For the ECEM project, the Brier^5^ and ROC skill scores were uses for this, and were calculated for each European country individually. These results are available on the ECEM Demonstrator, and summarised in Bett et al. (2018b).

### 3.3 Indirect forecasting of climate variables

So far, we have only considered ‘direct’ forecasting, in the sense of using one quantity output from a forecast model to predict the same quantity in observations, albeit via linear regression. However, a useful feature of the linear regression approach described above is that it offers a straightforward way to make ‘indirect’ forecasts: using one climate variable to predict another variable, possibly at a different location.

For example, in the scatter plot shown in Fig. 3, we could replace the variable on the horizontal axis with any other predictor from the forecast models. This could be the same meteorological variable, but measured over a larger area, to increase the skill: for example using the mean wind speed over an area covering the whole British Isles region, land and sea, to forecast the UK mean wind speed. This could be particularly important when forecasting for smaller regions or countries in Europe, as low levels of skill can often be improved by averaging over a larger area, if the wind speeds are sufficiently spatially correlated, by reducing the gridpoint-scale noise. The method then functions as a simple statistical downscaling technique.

**Fig. 5:**
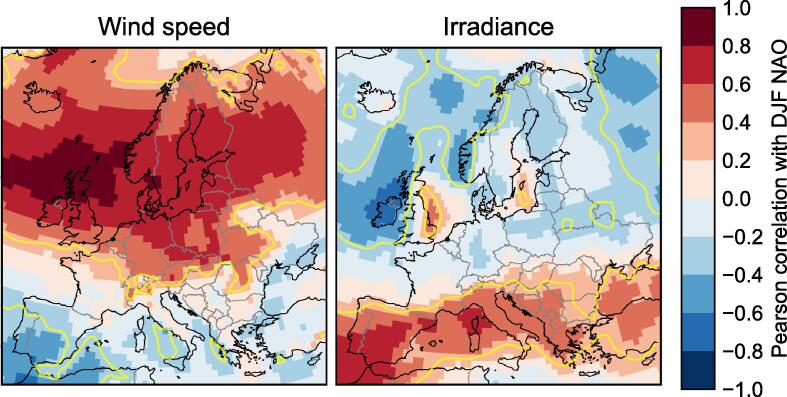
Maps of the correlation between the DJF NAO index and 10 m wind speed (left), and irradiance (right), using ERA-Interim data (winters 1979/1980 to 2015/2016 inclusive). Contours are included in yellow at r=±0.325, the notional threshold for significance over 37 years at the 5% level.

Another alternative is to use a larger-scale dynamical feature of the climate, such as the NAO, to forecast a local meteorological variable. It is well known that the NAO is well-correlated with many features of the northern and southern European winter climate, and we demonstrate the observed correlation of a simple NAO index^6^ with winter wind speed and irradiance in Fig. 5. If it can be skilfully predicted, then using the NAO index as the predictor can lead to more skillful forecasts of the target variable in many cases. Recent advances in seasonal climate prediction systems have demonstrated significant skill in forecasting the NAO (e.g. Scaife et al., 2014, Butler et al., 2016, Athanasiadis et al., 2017, Baker et al., 2018b), leading in turn to demonstrations of improved skill in other variables across Europe (e.g. Karpechko et al., 2015, Svensson et al., 2015, Clark et al., 2017, Baker et al., 2018a). A similar approach has been successfully applied to forecasts of rainfall in China (Bett et al., 2020).

Finally, as mentioned earlier, this formalism could also be used for forecasting quantities other than the mean value: a user might be more interested in the risk of some event, such as an extreme, occurring within the season. The details in these cases would be highly user-specific, but examples might include forecasting the number of low-wind days per season, or the number windstorms per season (Befort et al., 2019). Calculating this kind of counting statistic directly from the forecast model ensemble is likely to be noisier, and hence less skillful, than a seasonal mean. However, it might be possible in some situations to use the seasonal mean from the forecast system to predict the seasonal frequency of the event of interest, using observations of those frequencies in the regression. Thornton et al. (2019), in their study of seasonal forecasts of gas demand, provide an example of this situation. They found that the observed seasonal mean gas demand can be linearly related to atmospheric circulation indices from the forecast model. However, the number of high gas demand days per winter showed a non-linear relationship, with many seasons having no high-demand days. A similar result was found for forecasts of tropical cyclone landfall counts in China (Camp et al., 2020, Mitchell and Camp, 2021), where the initial system was improved by moving from a linear to a Conway–Maxwell–Poisson regression model. In other cases it might be preferable to transform the required variables first to linearize the relationship.

## 4 Forecasting wind and solar power generation

There is a clear need in many applications for detailed models to transform meteorological variables into energy variables. Short term (daily, hourly or less) forecasts of wind or solar power, based on weather forecasts, need to be highly accurate to allow the output of individual sites to be carefully managed (e.g. Giebel et al., 2011, De Felice et al., 2015, Haupt, 2018). Similarly, climatological risk studies, for example to allow financing for individual site development, or for planning future transmission/distribution grid requirements, can also require accurate transformations across timescales (e.g. Cannon et al., 2015, Bett et al., 2016, MacLeod et al., 2018). Indeed, the ECEM national-scale wind and solar PV data, which we use as ‘observations’ here, were developed on that basis.

**Fig. 6:**
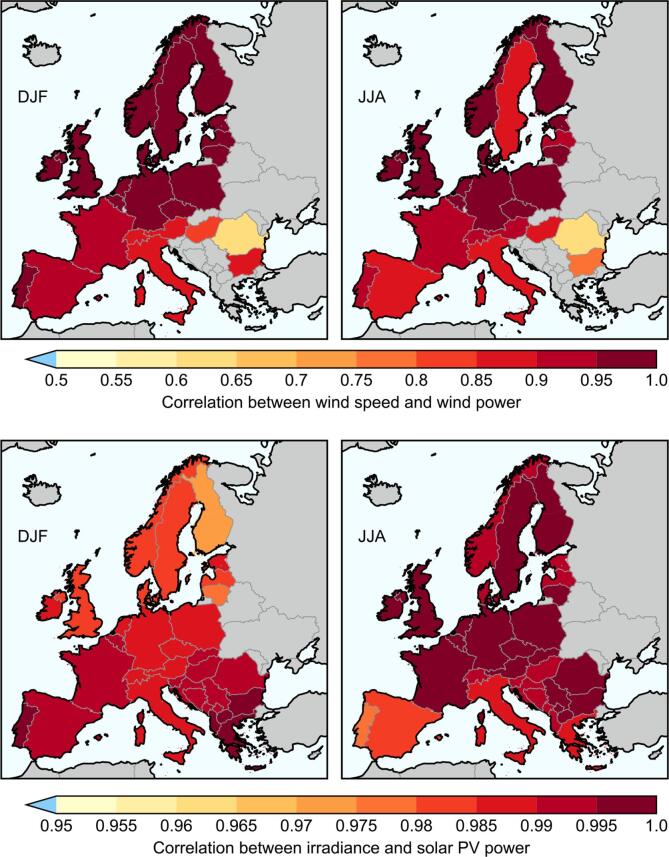
Maps of the correlation between the observed country-average climate variable and energy variable data, for DJF and JJA as labelled. Top: 10 m wind speed and wind power capacity factor. Bottom: irradiance and solar PV capacity factor. The ECEM climate and energy data is used in both cases. Note the different colour scales on the wind and solar panels.

However, the modest levels of skill (Fig. 1) and inherent uncertainties (Fig. 3, Fig. 4) of seasonal forecasts moderate our expectations of how accurate the forecasts on these temporal and spatial scales will be, suggesting that we can take a different approach. Fig. 6 shows the correlations between the observed climate and energy variables, at the seasonal-average, country-average scale. In the case of wind power, the correlation with mean wind speed for most countries is over 0.9, and apart from Romania (and in summer, Bulgaria) they all have r>0.8. In the case of solar power, all countries show correlations with irradiance greater than 0.97 (note the different colour scale).

**Fig. 7:**
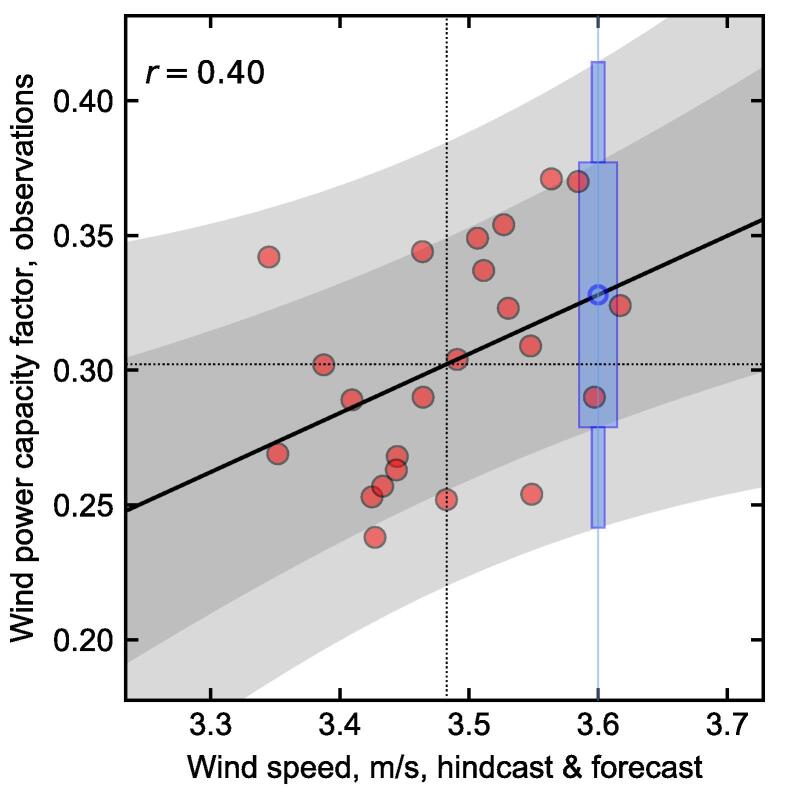
Scatter plot showing the relationship between observed winter wind power capacity factor in Finland, and the hindcast ensemble mean 10 m wind speed in Finland, using the Met Office system (as in Fig. 3). Other annotations are the same as in Fig. 3: The linear regression is shown as a black line, with the inner 75% and 95% of the prediction interval shown as grey shading. Mean values are shown as dotted lines. A hypothetical forecast point is shown in blue at $3.6\;m\;s^{-1}$, with boxes highlighting the prediction interval of the wind power capacity factor at that point.

The strength of these correlations means that, where there is skill in the underlying climate variable, we can use a simple linear regression to make a probabilistic forecast of the energy variable: just as in Fig. 3, but swapping out the observed climate variable on the vertical axis for the historical energy variable data. We demonstrate this explicitly in Fig. 7. The correlation skill of wind power forecast using the hindcast wind speed (0.40) is not significantly different at the 5% level to the wind speed forecast skill itself of 0.47 (as the data are based on the same set of years, we use Williams’s test, following Steiger, 1980).

It is worth emphasising some consequences of this, as it might be seen as going against common practice and understanding in energy meteorology:

• It is not beneficial to include the temperature dependence of solar PV generation: the correlation simply between solar capacity factor and irradiance alone is almost 1 everywhere.
• Scaling the wind speeds from the meteorological standard 10 m height to a more typical wind turbine hub height like 100 m is also likely to make no significant difference: Standard scaling procedures such as using a power law, whereby $U_{100m}={\left(100/10\right)}^{\alpha }U_{10m}$, do not affect the correlation (α is usually assumed to be constant); the scaling factor would automatically be captured by a linear regression forecast model.
• It is not necessary to use instantaneous wind speeds (or irradiance) at high temporal resolution and transform them through a power curve to obtain the wind power (or solar power), before seasonally averaging: there would be negligible improvement in skill over simply using the seasonal mean wind speed directly as the linear predictor of seasonal mean wind power.

**Fig. 8:**
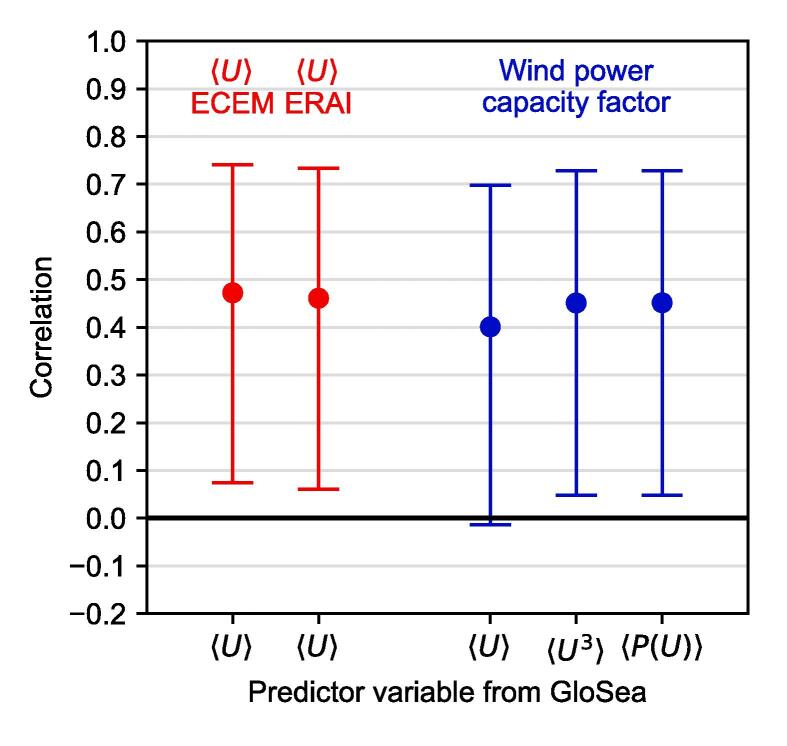
Examples of the impact of different forecast strategies on the correlation skill for Finland winter mean wind power capacity factor. The predictor variables are based on 6-hourly instantaneous 10 m wind speeds *U*, and we use angle brackets to indicate a seasonal mean. On the left (red), we show the skill of forecasting mean 10 m wind speed from both the ECEM and ERAI data sets using the GloSea5 seasonal mean wind speed as the predictor variable (cf. Fig. 3). On the right (blue), we use three different transformations of wind speed to forecast wind power capacity factor: The seasonal means of the wind speed itself (cf. Fig. 7), the cube of the wind speed, and the power-curve transformed wind speeds P(U). In all cases, we also show the 95% confidence intervals on the correlations using the Fisher z-test.

We demonstrate this last point explicitly in Fig. 8. Here we show the correlation skill of using 6-hourly instantaneous wind speeds from the GloSea5 system, transformed in different ways, to forecast seasonal mean wind power capacity factor. The simplest method, using the seasonal mean wind speed to forecast the seasonal mean wind power, has a correlation of 0.40, as shown in Fig. 7. The second method uses the seasonal mean of the cube of the instantaneous wind speeds. The third method transforms the instantaneous wind speeds into capacity factors directly using a wind turbine power curve (following Bett et al., 2016), before taking a seasonal mean. Both these latter cases result in correlations of 0.45. While there are small apparent numerical differences between the results of these different methods, when one considers the uncertainty on that skill it is clear that detailed, complex methods provide no detectable benefit over simply using the seasonal mean wind speed as the predictor variable. Indeed, Fig. 8 also shows that there is also no difference with the skill in forecasting the wind speed itself, from either the ECEM climate data set or ERA-Interim. This demonstrates the impact of the high correlations shown in Fig. 6, together with the modest skill shown in Fig. 1: detailed transformations are unlikely to result in improved skill. It is possible that detailed transformations could improve other metrics such as root mean square error, but again the differences are likely to be small compared to the overall forecast uncertainty.

A more plausible route to improved skill, in some cases, would be to use a larger-scale dynamical index as the climate variable predictor, as discussed in the previous section. This follows Palin et al. (2016), who demonstrated how the NAO can be used to forecast various quantities for the UK transport sector, such as the need for aircraft de-icing at Heathrow Airport.

The success of these kind of simplifications lies in the very strong correlations between the energy quantity of interest, and the climate-based predictand. This also determines the caveats on our findings: For example, these high climate–energy correlations do not occur universally. Bett et al. (2018a) and De Felice et al. (2018) demonstrated that electricity demand and hydroelectricity generation can both exhibit more complex relationships with the climate across Europe than solar PV and wind generation, showing strong correlations with the climate in some cases, and much weaker in others. They could therefore benefit from more careful modelling than a simple linear regression, or at least a more cautious case-by-case approach. Secondly, as discussed earlier, non-linear approaches might also be necessary if quantities other than a seasonal mean are required, such as the frequency of extreme events. Finally, there could be cases with existing or future forecast systems, where much higher levels of skill could be obtained, perhaps based on improved climate models, initialisation or ensemble construction. In that case, while a linear model would still work, it might be that a more sophisticated model relating the climate and energy variables could improve the skill further.

## 5 Optimisation from co-design

Much of what we have discussed so far in this paper has been achievable through the use of freely available data, for example from C3S, and indeed this itself represents a simplification compared to having to obtain data from individual providers in a variety of different formats. However, it is important to note that it is usually the case that the most optimal forecast services will be produced through a close co-development process: the climate service developer bringing in domain-specific expertise from both the energy (‘service user’) and climate (‘data producer’) sides.

The benefits of co-design and co-development in making forecast services more useful, and usable, by focusing them more on the practical needs of stakeholders, are well documented (e.g. Bruno Soares and Dessai, 2015, Bruno Soares and Dessai, 2016, Bruno Soares, 2017, Buontempo et al., 2017, Golding et al., 2017). It might be the case that the prospective user of the service needs forecasts issued at particular times of the year, or covering particular periods – where we have looked at forecasting DJF from November for example, a user might need longer lead times, or forecasts for financial quarters rather than meteorological seasons. It is important to understand that the skill in forecasting the particular season, at the particular lead time, will need to be assessed explicitly, rather than assuming that areas of high skill in one case will have similar skill in another case.

An important precondition of the linear regression approach we have described above is the availability of multi-decadal time series of the user’s quantity of interest. Although projects like ECEM provide much energy-sector time series data that can be applied to many cases, particular users are likely to require other specific quantities. It is unlikely that such data will exist covering the necessary time span, and even data over a shorter period might be commercially sensitive and unavailable publicly. This means that an additional modelling or calibration step might be required, following the approach taken by ECEM, to relate users’ recent energy data to longer-term climate variability. This additional modelling will bring its own uncertainties, which would also need to be assessed.

More optimal use of seasonal forecasting data can also be achieved though direct engagement with the providers of that data. They will have in-depth knowledge of the behaviour of their forecasting systems, and will be able to advise on ways to optimise their use. For example, there are now a wide range of seasonal forecast models, and model versions, available through the C3S Climate Data Store. While they are well documented, it requires a degree of expert judgement to assess whether/which different model ensembles can be pooled together, or if only particular models or versions should be used.

Another benefit of climate service developers engaging directly with climate data providers is that the service could benefit from research into more optimal post-processing of the model data. For example, Baker et al. (2018a) and Thornton et al. (2019) both demonstrate improvements in forecast skill from selecting appropriate large-scale predictors for their specific impact metrics, as discussed in Section 3.3. De Felice et al. (2018) and Stringer et al. (2020) demonstrate more complex post-processing used to derive the daily data needed for hydrological applications, while retaining the skillful signals from the larger-scale predictors.

## 6 Conclusions and summary

We have demonstrated the baseline levels of skill of seasonal forecasting systems available from C3S for seasonal-mean wind speeds and solar irradiance across Europe, at 1-month lead times, showing that the skill is patchy. Seasonal forecasts must therefore be used selectively and carefully.

We have described a simple method for producing calibrated probabilistic seasonal-mean forecasts for the cases where there is significant skill, based on the linear regression of the observational timeseries on the corresponding hindcast ensemble means. The hindcast variable can be different to the variable being forecast, and indeed skill might be improved in some cases by using a larger-scale climate predictor such as the NAO. Going further, the variable being forecast need not be a meteorological observable, but could be the energy metric required directly by the climate service recipient – thus providing a simple way of producing well-calibrated probabilistic forecasts of seasonal-mean wind and solar power generation potential.

This is possible because of the very high correlations we have demonstrated on seasonal mean, regional mean scales between wind power and wind speed, and between solar PV power and irradiance. In this context, and given the modest levels of skill available in the climate variables, there is likely to be negligible benefit to using more complex transformations to estimate these gross primary energy quantities, e.g. using high temporal resolution, or multiple variables – although these approaches remain critical in other energy–meteorological analysis and forecasting settings. The temporal or spatial scales at which a more complex approach might be necessary is an important area for future exploration, but is likely to be highly application-specific.

It is the country, seasonal and ensemble averaging that allows the linear regression method to work well, by reducing noise and pushing variables towards being normally distributed and linearly related. We emphasise, however, that this means that this approach will not be appropriate in all cases. For example, the number of extreme events per season is unlikely to be linearly related to a climate driver (Thornton et al., 2019), and in some use cases more sophisticated downscaling techniques might need to be developed if higher spatial or temporal resolution is required (e.g. De Felice et al., 2018, Stringer et al., 2020).

For many cases however – and together with the increased availability of seasonal forecasting data through initiatives like the C3S Climate Data Store and the ECEM Demonstrator tool – our results show how the process of developing useful seasonal forecasting climate services for wind and solar power can be greatly simplified. Further optimisation of the forecasts could also be possible, by drawing on the domain expertise of both the climate model data providers and the energy sector stakeholders, tailoring the service by balancing model capabilities and user needs. In all cases however, there is scope for much greater use of seasonal forecasts, aiming to reduce financial risks for the renewable energy sector, and improve energy security and energy system resilience more widely.

## CRediT authorship contribution statement

**Philip E. Bett:** Conceptualization, Methodology, Investigation, Software, Visualization, Writing – original draft, Writing – review & editing. **Hazel E. Thornton:** Conceptualization, Writing – review & editing, Supervision. **Alberto Troccoli:** Conceptualization, Writing – review & editing, Project administration. **Matteo De Felice:** Conceptualization, Methodology, Writing – review & editing. **Emma Suckling:** Conceptualization, Writing – review & editing. **Laurent Dubus:** Conceptualization, Methodology, Data curation, Writing – review & editing. **Yves-Marie Saint-Drenan:** Conceptualization, Methodology, Writing – review & editing. **David J. Brayshaw:** Conceptualization, Writing – review & editing.

## Declaration of Competing Interest

The authors declare that they have no known competing financial interests or personal relationships that could have appeared to influence the work reported in this paper.

## Figures and Tables

**Fig. A.9:**
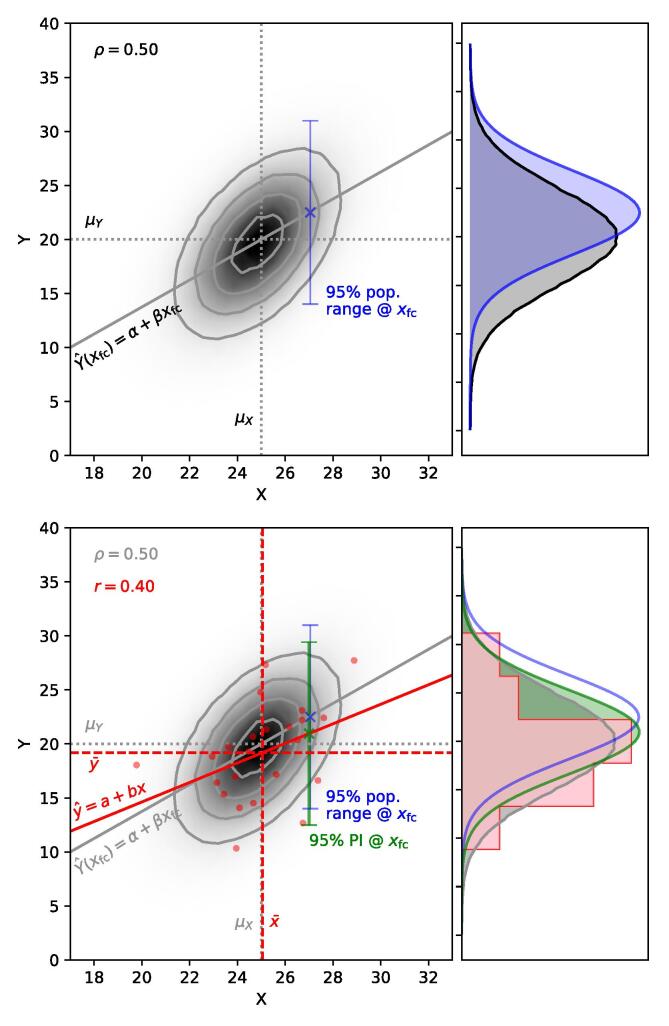
Diagram illustrating the mathematical terms described in the text. The upper panels illustrate the population statistics: the shading and contours show the bivariate normal distribution of *X* and *Y*. The linear regression is shown, and a forecast point is marked at $x_{\mathrm{fc}}$ with the 95% range of the population distribution at that point. On the right, the marginal distribution of *Y* is shown in grey, and the distribution of *Y* at the forecast point $x_{\mathrm{fc}}$ is in blue. The lower panels supplement these with a 23-point sample of the population (red points and annotations, with marginal distribution on the right). The 95% prediction interval at the forecast point is shown in green, as well as its distribution on the right. The population correlation ρ and the sample correlation *r* are labelled on the panels.

**Fig. A.10:**
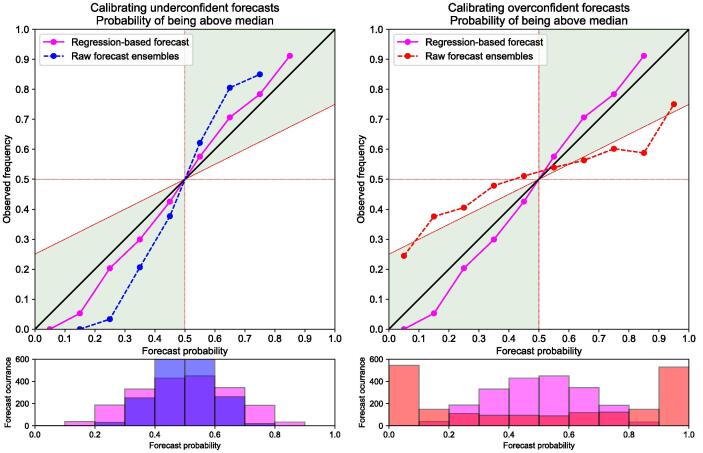
Reliability and sharpness diagrams for forecasting probabilities of an observation being above the climatological median. The region of positive Brier skill score is shaded in green, and the perfect reliability line (1:1) is shown in black. Left: The results from an underconfident (overdispersive) forecast ensemble (blue), with the results from forecasts produced using the linear regression method (magenta). Right: The results from an overconfident (underdispersive) forecast ensemble (red), with the linear regression results again in magenta (identical to the left-hand plot). All results uses the same artificial data for observations and the hindcast and forecast ensemble means.

## Appendix A The reliability of probabilistic forecasts produced by linear regression

Here we demonstrate that the linear regression method described in the text, using the hindcast ensemble means, necessarily calibrates the forecast probabilities within the limitation of the available data, if the hindcast is skillful. The description is based on Wilks (2020), and we refer the reader there for further details. Fig. A.9 illustrates the mathematical description.

The hindcast data (ensemble means from an initialised climate model) and observation-based data are samples from an underlying population joint distribution, which describes the relationship between the climate model and the real world in terms of the variables of interest. The hindcast is sampled from a predictor variable *X*, and the observations are sampled from a predictand variable *Y*. (We use these names to correspond to the *x* and *y* axes of a scatter plot such as in Fig. 3.) We assume that the population follows a bivariate normal distribution, with marginal normal distributions

(A.1) $$X∼N\left(\mu_{X},\sigma_{X}^{2}\right),$$

(A.2) $$Y∼N\left(\mu_{Y},\sigma_{Y}^{2}\right),$$

where μ and σ represent the population means and standard deviations respectively. The conditional distribution of observations *Y* given a ‘forecast’ $X=x_{\mathrm{fc}}$ is also a normal distribution:

(A.3) $$Y|x_{\mathrm{fc}}∼N\left(\mu_{Y|x_{\mathrm{fc}}},\;\sigma_{Y|x_{\mathrm{fc}}}^{2}\right)$$

(A.4) $$∼N\left(\mu_{Y}+\frac{\sigma_{Y}}{\sigma_{X}}\rho \left(x_{\mathrm{fc}}-\mu_{X}\right),\;\left(1-\rho^{2}\right)\sigma_{Y}^{2}\right),$$

where ρ is the correlation between *X* and *Y*. The mean of that conditional distribution is given by the linear regression of *Y* on *X* at that point,

(A.5) μY|xfc≡Y^(xfc)=α+βxfc,

where

(A.6) $$\alpha =\mu_{Y}-\beta \mu_{X},$$

(A.7) $$\beta =\rho \frac{\sigma_{Y}}{\sigma_{X}}\text{.}$$

The conditional distribution in Eq. (A.4) is the sampling distribution for the observation that occurs when the climate model (the ensemble mean forecast) produces the value $X=x_{\mathrm{fc}}$. If a forecast system is reliable, in the statistical sense, then of the times when an event is forecast with a given probability, it is observed to occur with a frequency equal to that probability. If we knew the population parameters, then the result of forecasting using linear regression would be exactly reliable: if our forecast system issued a value $x_{\mathrm{fc}}$, then the probability of observing a value of *Y is* simply the conditional distribution that describes the observed frequency, $Y|x_{\mathrm{fc}}$.

In reality however, we have a limited sample of *n* points (years) in both observations $y_{i}$, and hindcast $x_{i}$, for i=1…n. This adds uncertainty due to sampling variation, which we need to take into account. The linear regression we would derive from the *n* pairs of sample points is

(A.8) y^=a+bx,

such that the central prediction of an observation based on the predictor point $x_{i}$ is y^i=a+bxi. That regression model point y^i differs from the actual observation $y_{i}$ by an error $e_{i}$, such that yi=y^i+ei. The regression model parameters are given by

(A.9) $$a=\overset{¯}{y}-b\overset{¯}{x}$$

(A.10) $$b=\frac{\underset{i}{\sum }\left(x_{i}-\overset{¯}{x}\right)\left(y_{i}-\overset{¯}{y}\right)}{\underset{i}{\sum }{\left(x_{i}-\overset{¯}{x}\right)}^{2}}\equiv r\frac{s_{y}}{s_{x}}\text{.}$$

where the overbar represents the sample mean, *s* represents the sample standard deviation, and *r* is the sample correlation from the *n* points.

So, the actual conditional probability distribution that we could measure, for the observation that will occur after the climate model has produced the forecast point $x=x_{\mathrm{fc}}$ – i.e. the prediction interval – can be written as

(A.11) yfc∼Ny^fc,sfc2.

For the probabilistic forecasts made using this method to be reliable, then this has to match the true underlying conditional distribution of the observations given a forecast, Eq. (A.4).

Just as in Eq. (A.5), the mean in Eq. (A.11) is given by the linear regression, y^fc=a+bxfc. The sample variance corresponding to $\sigma_{Y|x_{\mathrm{fc}}}^{2}$ is the variance of observational points around the regression line, $s_{y|x}^{2}$, i.e. simply the variance of the errors,

(A.12) sy|x2≡se2=1n-2∑iyi-y^i2.

However, because the regression line here is itself an estimate, the prediction variance $s_{\mathrm{fc}}^{2}$ is bigger than $s_{y|x}^{2}$: we need to add a term for the sample variation in the estimate $\overset{¯}{y}$ of the observational mean $\mu_{Y}$ (equivalent to the error on the intercept estimate *a*); and a term that accounts for the error in the regression gradient *b*. Together, these terms give the required variance,

(A.13) $$s_{\mathrm{fc}}^{2}=s_{e}^{2}\left(1+\frac{1}{n}+\frac{{\left(x_{\mathrm{fc}}-\overset{¯}{x}\right)}^{2}}{\underset{i}{\sum }{\left(x_{i}-\overset{¯}{x}\right)}^{2}}\right)\text{.}$$

If the linear regression represents a very good fit, then $s_{e}^{2}$ will be small. Furthermore, if there is a large number of data points, then the second term (1/n, from estimating the regression intercept) will be small, and the third term will also be small (assuming $x_{\mathrm{fc}}-\overset{¯}{x}$ is a similar size to the other $x_{i}-\overset{¯}{x}$, then it is reduced by there being *n* such terms in the denominator): $s_{\mathrm{fc}}^{2}$ will therefore tend towards $\sigma_{Y|x_{\mathrm{fc}}}^{2}$ for large *n*.

Therefore, if the forecast system is significantly skillful and there is a genuine linear relationship between the observations and the climate model output, then the prediction interval will provide the best estimate of the conditional distribution of observations given a forecast value from the climate model – i.e., it will provide probabilistic forecasts that are well calibrated given the sampling uncertainty.

Recently, Yang et al. (2016) and Yang et al. (2018) have investigated the relationship between correlation and the reliability and resolution components of the Brier skill score, both empirically and theoretically. They found a clear relationship between the correlation and the resolution score, which measures the ability of a forecast system to resolve events into groups with different observed frequencies. However, they demonstrated that there is no clear relationship between the correlation and the reliability. This is not inconsistent with our reasoning here: Although we are relating correlation and reliability in some sense, it is the linear regression, rather than the correlation, that allows us to produce calibrated probabilities.

Fig. A.10 shows reliability/attributes diagrams (Wilks, 2020, Hsu and Murphy, 1986), that demonstrate our linear regression technique in calibrating an underconfident and an overconfident forecast system, through Monte Carlo simulation. We first sample 23 “hindcast ensemble mean” and “observation” points from a bivariate normal distribution. This corresponds to the number of years, or seasonal means, in the data sets, and was chosen to match the data in Fig. 3. We set the population correlation (skill) ρ=0.50, and set the population mean and standard deviation of the observations to be 20 and 5 respectively; and for the hindcast, 25 and 2 respectively.^7^ We calculate the linear regression on the joint distribution of those sample points. We then produce 2000 new “forecast ensemble mean” points, and the corresponding new “observations” that would occur afterwards, by again sampling from that original bivariate normal distribution.

The reliability diagrams show the relationship between forecast probabilities and observed frequencies for forecasts of above-median conditions. For the first reliability diagram, we produce a 1000-member overdispersive (underconfident) ensemble for each of the 2000 forecast ensemble means, using an ensemble standard deviation of 9 (i.e. bigger than the standard deviation of the observations and the hindcast ensemble-means). The reliability diagram from these forecast ensembles demonstrate that the forecast system is underconfident: the line is too steep. The sharpness diagram also shows that the forecast ensemble tends to produce forecasts around the climatological frequency of 0.5.

We then apply the linear regression to each forecast ensemble mean to give central estimates and prediction intervals. We can then test the reliability of these predictions by sampling the prediction intervals 1000 times (like the original forecast ensembles). This indeed shows well-calibrated probabilities: the line is closer to the 1:1 diagonal. The sharpness is also improved, with the forecasts distributed over a wider range of probabilities.

We have also repeated this for an overconfident forecast ensemble, using an ensemble standard deviation of 1 (smaller than the observed and hindcast ensemble-mean standard deviations): here, the reliability line is too shallow, and the sharpness diagram shows a tendency to forecast extreme probabilities. The calibrated line based on the regression of the ensemble means is the same as the underconfident case, by construction.

It is important to note that the issue of having a limited sample of data with which to construct the linear regression, also applies to any other calibration method. Using the prediction intervals from a linear regression offers a robust way of taking that sampling uncertainty into account as part of the calibration process.

## Footnotes

2 The C3S Climate Data Store can be accessed at https://cds.climate.copernicus.eu

3 Offshore wind power data is now available from the C3S Energy operational service, athttps://climate.copernicus.eu/operational-service-energy-sector.

4 http://ecem.wemcouncil.org.

5 Note that the CRPS corresponds to the Brier score with all possible values used as forecast categories

6 We use the simple difference between the mean sea level pressure in a southern box around the Azores ($28^{\circ }\;W$–$20^{\circ }\;W,36^{\circ }\;N$–$40^{\circ }\;N$) and a northern box around Iceland ($25^{\circ }\;W$–$16^{\circ }\;W,63^{\circ }\;N$–$70^{\circ }\;N$) for our NAO index, following Dunstone et al. (2016).
